# Two-dimensional semiconductors pave the way towards dopant-based quantum computing

**DOI:** 10.3762/bjnano.9.249

**Published:** 2018-10-12

**Authors:** José Carlos Abadillo-Uriel, Belita Koiller, María José Calderón

**Affiliations:** 1 Materials Science Factory, Instituto de Ciencia de Materiales de Madrid (ICMM-CSIC), Sor Juana Inés de la Cruz 3, 28049 Madrid, Spain; 2 Instituto de Física, Universidade Federal do Rio de Janeiro, Caixa Postal 68528, Rio de Janeiro, RJ 21941-972, Brazil

**Keywords:** two-dimensional (2D) materials, dopants, qubits, quantum computing

## Abstract

Since the proposal in 1998 to build a quantum computer using dopants in silicon as qubits, much progress has been made in the nanofabrication of semiconductors and the control of charge and spins in single dopants. However, an important problem remains unsolved, namely the control over exchange interactions and tunneling between two donors, which presents a peculiar oscillatory behavior as the dopants relative positions vary at the scale of the lattice parameter. Such behavior is due to the valley degeneracy in the conduction band of silicon, and does not occur when the conduction-band edge is at *k* = 0. We investigate the possibility of circumventing this problem by using two-dimensional (2D) materials as hosts. Dopants in 2D systems are more tightly bound and potentially easier to position and manipulate. Moreover, many of them present the conduction band minimum at *k* = 0, thus no exchange or tunnel coupling oscillations. Considering the properties of currently available 2D semiconductor materials, we access the feasibility of such a proposal in terms of quantum manipulability of isolated dopants (for single qubit operations) and dopant pairs (for two-qubit operations). Our results indicate that a wide variety of 2D materials may perform at least as well as, and possibly better, than the currently studied bulk host materials for donor qubits.

## Introduction

Defects are a crucial concept in semiconductor technology as they provide proper carriers to intrinsically insulating semiconductors. Dopants constitute the basis for transistor operations. The miniaturisation of these devices has moved defects to the forefront of research, as their number and location may affect device performance and reproducibility [1]. Few-donor specific configurations were explored by Kane [2] in his Si quantum-computer proposal, based on an array of donors in which each of them acts as a spin qubit. This, in principle, leads to a scalable quantum computer and would be compatible with the existing Si-based transistor industry. For spin qubits, Si has the additional advantage of sustaining very long spin-coherence times, up to seconds for isotopically purified Si [3].

The effort to understand single-donor behavior has led to significant raise of expertise on the manipulation and quantum control of states bound to donors in the last few years [4–8]. One problem of using donors in Si for qubits, as proposed by Kane [2], is that interference among the multiple degenerate Si conduction band minimum states leads to a sensitive and oscillatory behavior of tunnel [9] and exchange [10] coupling of electrons bound to pairs of donors as the relative positions of the donors vary. Although no oscillatory behavior is expected for coplanar dopant pairs relative to (001) planes under tensile stress, any individual dopant deviation in the *z*-direction restores the oscillations [11]. This problem can be deterrent to the implementation of quantum computing in Si due to the relative lack of control about the exact position of dopants in the bulk. Alternative proposals suggested to overcome this difficulty include hybrid dopant–quantum dot structures [12–13], a charge–spin hybrid qubit [14], optical manipulation [15] and dipole coupling with electrons [16] or holes [17–18].

**Figure 1:**
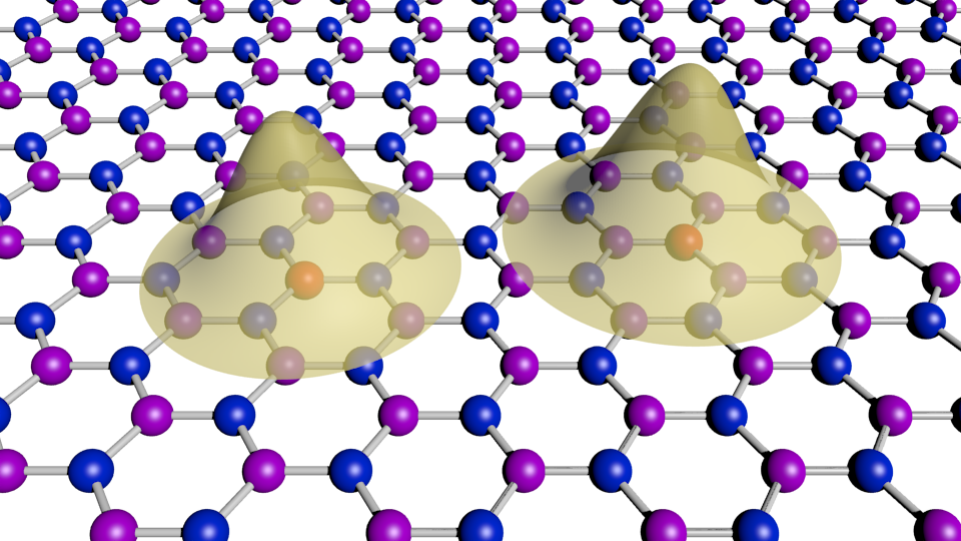
Schematic representation of the electronic distributions around two donors (represented in red) in a 2D material. Many of the 2D materials currently under study have a graphene-like crystal structure (two sublattices represented by blue and violet dots). Here we explore, within an effective mass approach, the possibility of using these donors to implement spin-qubits. Some of those 2D structures may present advantages over bulk (3D) semiconductor hosts.

Here we propose an alternative that relies on two-dimensional (2D) semiconductor materials instead of bulk Si as host material. A precise positioning of donors on a surface may be simpler than in the bulk, because it only requires control over two coordinates, avoiding the *z*-component uncertainties, see Figure 1. More importantly, quite a few 2D materials present the conduction band minimum at the Γ point [19–20], naturally reducing the required donor positioning accuracy, as no oscillatory exchange and tunnel couplings are predicted in this case.

The family of 2D materials comprises an increasing number of elemental and compound semiconductors [21–23]. Many have been experimentally isolated already, as research activity in this area raises. In the case of non-metallic behavior, their band gaps range from millielectronvolts to a few electronvolts. They can also be stacked in van der Waals heterostructures [22,24–25] favoring miniaturization and device integration. Incorporation of dopants affects the properties of isolated or stacked monolayers [26–27], as they do in bulk systems. Here we explore doping in the very-low-density limit such that electrons can be bound to single donor atoms and pairs of donor atoms in a 2D environment in the context of quantum computation.

This manuscript is organized as follows: In the following section, we give general arguments to estimate parameters characterizing different 2D materials, general trends and approximations. After that, results and discussion on the potential of dopants in 2D materials to define qubits are presented. We end the manuscript with the conclusions.

## Relevant Parameters and Formalism

We analyze the stability of bound states in single dopants and the coupling between pairs of donors in a 2D semiconductor host using an effective mass approach (EMA). We consider single donors and donor pairs in 2D. Within EMA, the discrete crystal structure of the device is described by a continuum characterized by the effective mass *m*_eff_ and the dielectric screening ε of the host materials. In atomic units, the binding energy in 2D is larger than in 3D for particular values *m*_eff_ and ε. Defining the effective Rydberg constant as


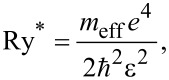


the binding energy of the electron bound to a single dopant in 3D is 
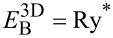
 while in 2D it is 
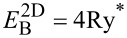
. Similarly, defining


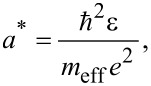


the respective Bohr radii are *a*^3D^ = a^*^ while *a*^2D^ = a^*^/2. The values of the effective units depend on *m*_eff_ and ε: Ry^*^ = 13.6*m*_eff_/ε^2^ eV and a^*^ = 0.529ε/*m*_eff_ Å.

The gap and the effective masses of different semiconducting 2D materials have been estimated from band-structure calculations [19,28]. Some gaps are also known experimentally from transport and optical measurements [21–23]. The size and nature (direct or indirect) of the gap depends on the number of layers [29], the distance between them [28], and the nature of the substrate or atomic reconstructions [30]. Properties of these materials may vary and eventually be tuned by an electric field, for instance, in the case of buckled silicene and germanene [31].

There is much less information on the dielectric screening of 2D materials, which also depends on the substrate and environment. It has been calculated only for a few cases (for instance, MoS_2_[32] or h-BN [33]) and the experimentally reported values lie in a wide range [32]. Typically, the dielectric constant of monolayer materials is expected to be smaller than their 3D counterparts, as their screening capabilities are reduced at low dimensionality [29,33]. All this variability would give rise to an expected dispersion of the binding energy of dopants depending on external factors. Accordingly, it has been shown, using first principles calculations in transition-metal dichalcogenides, that dopants can be tuned from deep to shallow by using different substrates [34]. This modulation of ionization energy has been studied in the context of achieving p-type/n-type doping for transistor-like devices, but it certainly remains relevant for the donor quantum manipulation proposed here.

Another important issue to take into account is the fact that in 2D systems the dielectric function is non-local. As discussed in [35], it may be written as ε(**q**) = 1 + 2πα|**q**|, with α being the polarizability. Hence, for the description of the impurity potential we should take into account the dependence of the screening ε on the distance from the donor. However, it has been recently found that the effect of a non-local dielectric function can be reproduced by a dielectric constant given by its average within the radius of the wave-function [36], dramatically simplifying the energy calculations. In [36], this simplification has been proven to be accurate for the calculation of the exciton binding energies of 51 transition-metal dichalcogenides. We follow the same approach here, taking a constant ε to estimate the binding energies.

**Figure 2:**
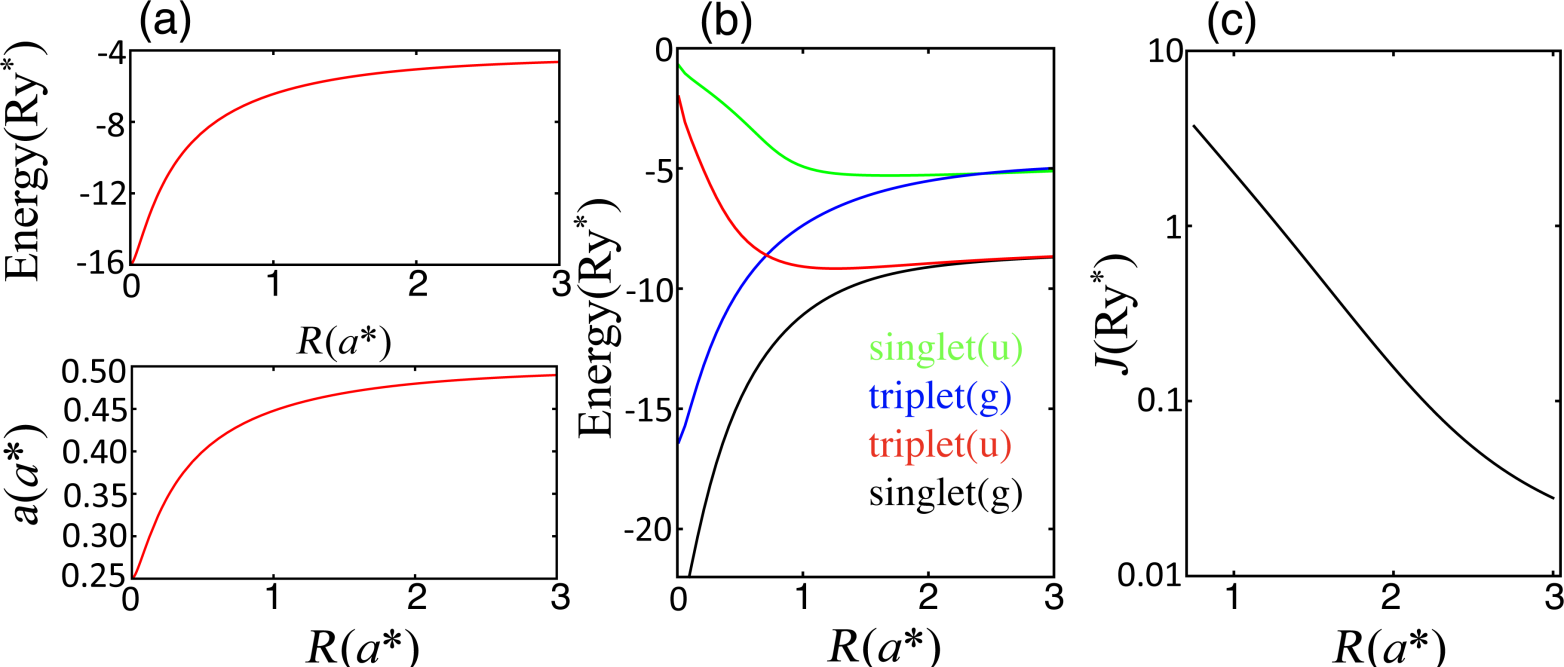
(a) Bohr radii 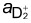 and energy for one electron bound to a donor pair 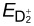 as a function of *R*. For *R* = 2a^*^, 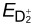 = −5.06 Ry^*^ and 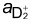 = 0.48a^*^. Assuming ε = 5 and using the effective masses shown in Table 1, 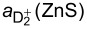 = 6.8 Å and 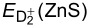 = −515 meV, 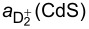 = 7.6 Å and 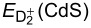 = −460 meV, 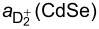 = 10 Å and 
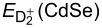
 = −350 meV, 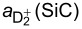 = 1.96 Å and 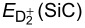 = −1.77 eV. Using *m*_eff_ and ε for MoS_2_ and h-BN in Table 1, we obtain 
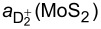
 = 2.2–2.7 Å and 
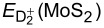
 = −1.59 eV, and 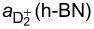 = 0.5 Å and 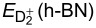 = −15 eV. (b) Energies for two electrons bound to a donor pair as a function of the inter-donor distance *R*, see Supporting Information File 1 for the definition of the wave function. (c) Exchange *J* in effective units as a function of the separation between donors. For *R* = 2a^*^, *J* = 0.156 Ry^*^. For this distance, assuming ε = 5 and using the effective masses in Table 1, *J*_ZnS_ = 16 meV, *J*_CdS_ = 14 meV, *J*_CdSe_ = 11 meV, *J*_SiC_ = 55 meV. Using *m*_eff_ and ε for MoS_2_ and h-BN in Table 1, we get 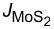 = 50–60 meV and *J*_h−BN_ = 467 meV.

We adopt isotropic envelopes, simplifying the calculations while keeping the physical picture [37]. In this approximation, the 2D bound state of hydrogen is


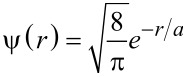


with *a* = a^*^/2. For a single electron bound to a dopant pair 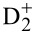, see Supporting Information File 1, the wave function radius and the binding energy are functions of the inter-donor separation *R*, as shown in Figure 2a. For *R* = 0 one gets a He-like positive ion, He^+^, with binding energy 16Ry^*^ and a Bohr radius a^*^/4. For very large *R*, we obtain the result of hydrogen, as the electron would only be bound to one of the dopants. For two electrons bound to a dopant pair D_2_, at least two variational parameters are required, see Supporting Information File 1.

**Table 1:** Effective masses and band-gap energies of selected 2D materials. ZnS, CdS, CdSe and SiC have a direct band gap at the Γ point. h-BN and MoS_2_ have one at the *K* point. In the literature, values for the dielectric constants (mostly calculated) can only be found for a few materials and, as discussed in the text, they depend on external conditions. Therefore, we consider the dielectric constant as a parameter. Unless otherwise stated, the data are taken from [28].

material	effective mass (*m*_eff_)	band-gap energy (eV)	dielectric constant (ε_0_)

ZnS	0.187	2.58–4.5	—
CdS	0.167	1.72–3.23	—
CdSe	0.127	1.30–2.47	—
SiC	0.645	2.55–3.63	—
MoS_2_	0.37 [38]–0.45 [39]	1.3–1.9 [21]	4 [32]
h-BN	1.175	5.9 [21]	2.31 [33]

## Results and Discussion

**Figure 3:**
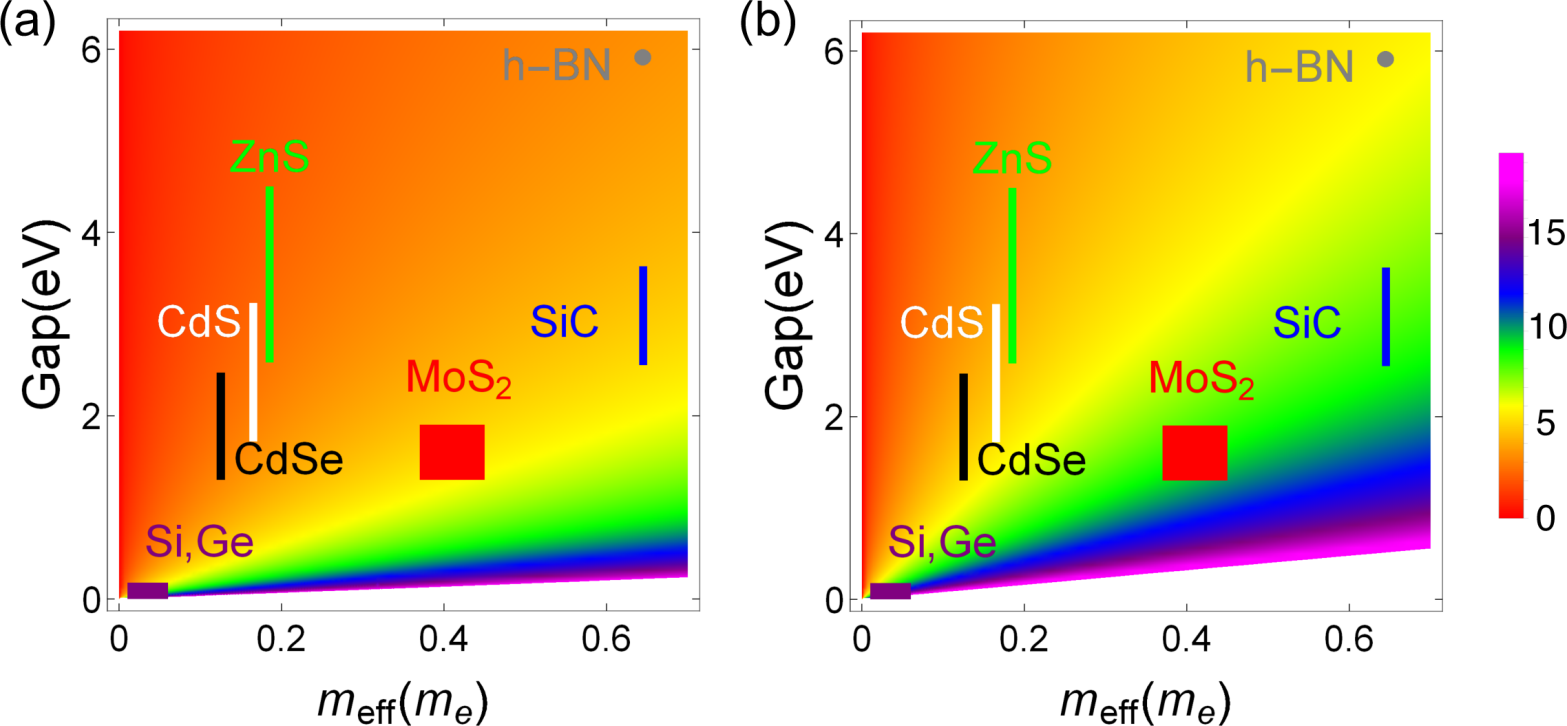
Minimum dielectric constant that guarantees the existence of bound states and the validity of EMA for isolated dopants (a) and dopant pairs separated by *R* = 2a^*^ (b). We expect the known values of ε to be in the yellow–orange–red region of the map, which encloses many of the analysed materials. The range of values for masses and gaps available in the literature and summarised in Table 1 are shown by the extended symbols next to the corresponding material composition.

The EMA is appropriate to describe shallow states in semiconductors, thus the band gap energy of the considered material has to be much larger than the binding energies *E*_B_. In order to implement this condition, we consider the generally unknown dielectric constant as a free parameter and estimate its minimum value required for the binding energy to fulfill the condition *E*_B_ *< E*_g_/2 as a function of the band-gap energy *E*_g_ and the effective mass on the conduction band, see Figure 3. Based on the known values of ε, an estimate ε ≤ 5 seems reasonable. This corresponds in the rainbow color code in Figure 3 to the yellow–orange–red region of the plots. For shallow donors, the condition would actually be 
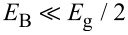
, and hence the points would be blue-shifted, meaning larger values of ε.

In order to put our results in the context of available 2D materials, we also include in Figure 3 data from Table 1. The yellow–orange–red region of the plots includes various 2D materials that, in terms of energetics of the bound states, could host shallow donor states. The condition is somewhat more restrictive for donor pairs as the corresponding binding energy is enhanced for the short inter-donor distances (*R* = 2a^*^) considered. For larger values of *R*, the small ε region is expanded. In general, in this yellow–orange–red region we find the first three materials in Table 1, and possibly silicene and germanene if their band gap energies were suitably enhanced.

In order to estimate binding energies and Bohr radii, we assume ε ≈ 5 for the first three materials in Table 1. With this value, the Bohr radii for electrons bound to single dopants would be between 1 and 2 nm, which is comparable to the corresponding values for 3D Si [37]. Consistently, the binding energies are similar to those in 3D Si, with values ranging from 70 to 100 meV. The last three materials in Table 1 would be much more confined, with Bohr radii within a few angstroms and energies up to few electronvolts.

Although EMA is not designed to treat large *E*_B_ values, it is certain that the wave functions in this limit are more confined (smaller effective Bohr radii). This is a desirable property in terms of isolation of the qubit and robustness against decoherence processes.

Now we turn to the conditions for two-qubit operations. In the original Si quantum computer proposal [2], two-qubit operations are driven by exchange gates, i.e., exchange coupling *J* pulses between electrons bound to neighboring donors. For two electrons bound to a single donor there are two low-energy levels well separated from the next excited state, one singlet and one triplet, which allows to map the lower-energy-states problem to the Heisenberg spin-1/2 Hamiltonian. For two electrons bound to a donor pair, there are four possible orbital states (see Supporting Information File 1). We label the expectation values of the Hamiltonian for these states in increasing order *E*_1_, *E*_2_, *E*_3_ and *E*_4_, and assign a spin hamiltonian to this problem if 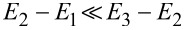 such that only the two lowest levels are relevant at low temperatures, and the spin-1/2 Hamiltonian may be defined as for a two-level system. It can be shown that the two lowest levels are a singlet and a triplet state (Figure 2a). Figure 2c shows *J*, the difference between the lowest singlet and triplet levels, as a function of *R* in a physically accessible range of inter-donor distances. For *R* = 2a^*^, *J* = 0.156 Ry^*^. For ε ≈ 5 and inter-donor separation *R* = 2a^*^, the exchange values cover a wide range, 15 meV *< J*(*R* = 2a^*^) *<* 100 meV, for the materials in the yellow–orange–red region in Figure 3. With these *J* values one would perform very fast (about 10^−14^ s) manipulations for 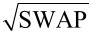 operations. If coherence times in 2D are about the same as in 3D, this would allow for a large number of operations within coherence times. *J* can be strongly enhanced in materials with larger binding energies but, in this case, sub-nanometer inter-dopant distances would be required, demanding a very high accuracy of the placement of gates on top and between donors. Note that most of the materials considered in Table 1 have a non-degenerate conduction-band minimum at *k* = 0, implying that the exchange coupling in Figure 2c would not oscillate as a function of the relative position of a donor pair. This should reduce the technological demands on donor positioning.

## Conclusion

The variability of binding energies as a function of the chemical composition, substrate and number of layers, opens up a wide range of possibilities for the potential use of 2D materials to host donors where electrons may serve as qubits. We distinguish 2D materials that support shallow states with binding energies and Bohr radii comparable to *P* in Si, and those that support stronger confinement. Each group of materials could serve different purposes with shallower states more suitable for manipulation and deeper ones for storage. The synergy among different experimental techniques for dopant positioning in 3D semiconductors, combined with recent advances in 2D materials-based electronics and multilayered architectures control, provide key technical tools for the practical implementation of donor-based spin qubits. Finally, some of the considered semiconductor 2D materials have no valley degeneracy, simplifying the experimental requirements of a scalable quantum computer.

## Supporting Information

Details on the variational approach used to calculate the different wave functions.

File 1 Variational wave functions.
