# Supplementary material for: Two-dimensional semiconductors pave the way towards dopant-based quantum computing
Source: Beilstein J Nanotechnol. 2018 Oct 12;9:2668–73. doi: 10.3762/bjnano.9.249 (PMC6204835; doi:10.3762/bjnano.9.249)
Supplement: File 1 — Variational wave functions. [file Beilstein_J_Nanotechnol-09-2668-s001.pdf]

Supporting Information

for

Two-dimensional semiconductors pave the way towards dopant-based quantum computing

José Carlos Abadillo-Uriel*[1], Belita Koiller[2] and María José Calderón[1]

Address: [1]Materials Science Factory, Instituto de Ciencia de Materiales de Madrid (ICMM-CSIC), Sor Juana Inés de la Cruz 3, 28049 Madrid (Spain) and [2]Instituto de Física, Universidade Federal do Rio de Janeiro, Caixa Postal 68528, Rio de Janeiro, RJ 21941-972, Brazil

Email: José Carlos Abadillo-Uriel - garciaabadil@wisc.edu

* Corresponding author

Variational wave functions

We are interested here in the ground-state properties of one or two electrons bound to single donors or donor pairs for which a variational scheme is appropriate. For non-degenerate conduction band edges, the rescaled atomic H ground state is a good trial wave function for one-donor situations. In case of two donors, a properly symmetrized combination of hydrogenic orbitals centered at each donor is a reasonable choice for a trial wave function. For example, for the molecular ion D_2^+ ground state we take the trial form $\Psi = N\left(e^{-r_{\mathrm{A}}/a'} + e^{-r_{\mathrm{B}}/a'}\right)$ where r_{A} and r_{B} represent the distance of the electron from the donors A and B and a' is a variational parameter, chosen to minimize the expected value of the energy. For the neutral molecule D_2 we take the combinations

$$\Psi^{g}_{\mathrm{singlet}}(\mathbf{r}_1,\mathbf{r}_2) = N[(e^{-\alpha r_{1\mathrm{A}}-\beta r_{2\mathrm{B}}} + e^{-\alpha r_{2\mathrm{A}}-\beta r_{1\mathrm{B}}}) + (e^{-\alpha r_{1\mathrm{B}}-\beta r_{2\mathrm{A}}} + e^{-\alpha r_{2\mathrm{B}}-\beta r_{1\mathrm{A}}}) + e^{\mathrm{i}\phi}(e^{-\alpha(r_{1\mathrm{A}}+r_{2\mathrm{B}})} + e^{-\alpha(r_{2\mathrm{A}}+r_{1\mathrm{B}})}) + e^{\mathrm{i}\theta}(e^{-\beta(r_{1\mathrm{A}}+r_{2\mathrm{B}})} + e^{-\beta(r_{2\mathrm{A}}+r_{1\mathrm{B}})})] \quad \text{(S1)}$$

$$\Psi^{u}_{\mathrm{singlet}}(\mathbf{r}_1,\mathbf{r}_2) = N[(e^{-\alpha r_{1\mathrm{A}}-\beta r_{2\mathrm{B}}} + e^{-\alpha r_{2\mathrm{A}}-\beta r_{1\mathrm{B}}}) - (e^{-\alpha r_{1\mathrm{B}}-\beta r_{2\mathrm{A}}} + e^{-\alpha r_{2\mathrm{B}}-\beta r_{1\mathrm{A}}})] \quad \text{(S2)}$$

$$\Psi^{g}_{\mathrm{triplet}}(\mathbf{r}_1,\mathbf{r}_2) = N[(e^{-\alpha r_{1\mathrm{A}}-\beta r_{2\mathrm{B}}} - e^{-\alpha r_{2\mathrm{A}}-\beta r_{1\mathrm{B}}}) + (e^{-\alpha r_{1\mathrm{B}}-\beta r_{2\mathrm{A}}} - e^{-\alpha r_{2\mathrm{B}}-\beta r_{1\mathrm{A}}})] \quad \text{(S3)}$$

$$\Psi^{u}_{\mathrm{triplet}}(\mathbf{r}_1,\mathbf{r}_2) = N[(e^{-\alpha r_{1\mathrm{A}}-\beta r_{2\mathrm{B}}} - e^{-\alpha r_{2\mathrm{A}}-\beta r_{1\mathrm{B}}}) - (e^{-\alpha r_{1\mathrm{B}}-\beta r_{2\mathrm{A}}} - e^{-\alpha r_{2\mathrm{B}}-\beta r_{1\mathrm{A}}}) + e^{\mathrm{i}\phi}(e^{-\alpha(r_{1\mathrm{A}}+r_{2\mathrm{B}})} - e^{-\alpha(r_{2\mathrm{A}}+r_{1\mathrm{B}})}) + e^{\mathrm{i}\theta}(e^{-\beta(r_{1\mathrm{A}}+r_{2\mathrm{B}})} - e^{-\beta(r_{2\mathrm{A}}+r_{1\mathrm{B}})})] \quad \text{(S4)}$$

α and β are the variational parameters. These variational wave functions correspond to the four lowest bound states. Only the orbital part is explicitly given. The spin part is inferred by symmetry and is indicated by the label singlet or triplet. The combination of different terms is included to preserve the symmetry (symmetric: gerade or antisymmetric: ungerade) under reflection A↔B. The undetermined relative phases θ and ϕ, included to allow for the most generic wave functions that fulfill the symmetry of H_2, are found to be both zero in the minimization process.
